# Case report: A clinical case study of six patients with *Chlamydia psittaci* pneumonia

**DOI:** 10.3389/fcimb.2023.1084882

**Published:** 2023-02-24

**Authors:** Jinmeng Dai, Xue Lian, Juanfen Mo, Xiaosi Li, Weiqiang Mo, Haiqin Wang, Jianping Jiang

**Affiliations:** ^1^ Department of Respiratory Medicine, The Second Affiliated Hospital of Jiaxing University, Jiaxing, Zhejiang, China; ^2^ Department of Respiratory Medicine, Jiashan County Yaozhuang Town Health Centre, Jiaxing, Zhejiang, China; ^3^ Department of Respiratory Medicine, Bengbu Medical College, Bengbu, Anhui, China; ^4^ The Key Laboratory, The Second Affiliated Hospital of Jiaxing University, Jiaxing, Zhejiang, China; ^5^ Clinical Laboratory, The Second Affiliated Hospital of Jiaxing University, Jiaxing, Zhejiang, China

**Keywords:** psittacosis, chlamydia, pneumonia, mNGS, doxycycline

## Abstract

**Objective:**

To investigate the clinical features, diagnosis, and treatment of *Chlamydia psittaci* (*C. psittac*i) pneumonia.

**Methods:**

We retrospectively analyzed the clinical data of six patients with *C. psittaci* pneumonia who were admitted to the Division of Pulmonary and Critical Care Medicine of the Second Hospital of Jiaxing from December 2021 to September 2022.

**Results:**

All patients reported a fever and other accompanying symptoms, including cough (5/6), chest tightness (1/6), fatigue (2/6), and headache (1/6). Laboratory results showed that all patients had high levels of C-reactive protein (CRP≥70 mg/L), procalcitonin (PCT; 2 patients with PCT levels ≥0.5 ng/L), and erythrocyte sedimentation rate (ESR). Lactate dehydrogenase (LDH) and aspartate aminotransferase (AST) levels were elevated in 3/6 and of 2/6 patients, respectively. Chest computed tomography (CT) of most patients showed patchy, high-density shadows with partial consolidation, accompanied by air bronchogram signs and pleural effusion. Six patients were diagnosed with *C. psittaci* pneumonia using metagenomic next-generation sequencing (mNGS). They showed favorable outcomes following immediate adjustment of the regimen to doxycycline-based therapy and hydration, nutrition, and other follow-up treatments. In the imaging findings obtained at one-two month, the lesions were completely cleared, suggesting a favorable prognosis.

**Conclusion:**

Patients with *C. psittaci* pneumonia commonly present sepsis and rapidly progressing disease. Early diagnosis is critical for *C. psittaci* pneumonia using mNGS, which can lead to favorable prognoses *via* immediate adjustment therapies.

## 1 Introduction

*C. psittaci* pneumonia, whose causative agent is the Gram-negative and obligate intracellular bacterium *C. psittaci* (Kong et al., 2021), is commonly transmitted *via* close contact with infected birds (such as parrots and poultry) or inhalation of aerosols from nasal secretions and dust from feces or feathers (Chen et al., 2022). *C. psittaci* pneumonia cases constitute approximately 1% of all community-acquired pneumonia (CAP) cases (Zhang A. et al., 2022). Owing to the non-specificity of its clinical manifestations, early diagnosis is difficult, and a rapid progression of the disease is observed in patients (Zhao et al., 2022). Additionally, antibiotics can promote the development of a severe and life-threatening disease. With the development and wide applicability of metagenomic next-generation sequencing (mNGS), the pathogenesis underlying *C. psittaci* pneumonia has been gradually elucidated (Teng et al., 2021). To better understand the clinical characteristics of patients with *C. psittaci* pneumonia, this study analyzed the clinical data of six patients who were admitted to the Second Hospital of Jiaxing in the last ten months.

## 2 Subjects and methods

### 2.1 Subjects

We identified six patients with pneumonia who were hospitalized at the Second Hospital of Jiaxing from December 2021 to September 2022 and showed positive mNGS results for *C. psittaci*. Demographic characteristics, including age, sex, occupational history, and history of bird exposure, were collected. Clinical manifestations, signs (maximum temperature and clinical symptoms), and laboratory and imaging findings were also recorded. This study was approved by the ethical committee of the Second Hospital of Jiaxing (approval number JXEY-2022SW067) and was in accordance with the ethical standards formulated in the Declaration of Helsinki. All patients whose clinical data were collected provided written informed consent and signed informed consent forms.

### 2.2 Diagnostic criteria

The diagnosis was confirmed *via* bronchoalveolar lavage fluid (BALF) samples collected from all patients based on the presence of *C. psittaci* sequences determined by mNGS.

### 2.3 Laboratory and imaging studies

White blood cell (WBC) count, neutrophil percentage, hypersensitive C-reactive protein (hCRP) and procalcitonin (PCT) levels, erythrocyte sedimentation rate (ESR) along with the levels of alanine aminotransferase (ALT), aspartate aminotransferase (AST), lactate dehydrogenase (LDH), and creatine kinase (CK) were recorded on the day of admission.

All patients underwent lung computed tomography (CT) prior to admission. The distribution, number, and type of lesions were described separately. The CT images were analyzed and characterized based on the following imaging features: ground-glass opacity, consolidation shadow, air bronchogram sign, reversed halo sign, centrilobular nodules, vacuole sign, lymphadenectasis, and pleural effusion.

BALF samples were obtained for smear and culture analysis. Samples were also subjected to sequencing by mNGS technology, which was performed *via* BGI2000 (More BioMedical, Hangzhou).

### 2.4 Treatment and outcomes

The types of antibiotics were recorded using a BGISEQ-2000 sequencing platform before and after the diagnosis of *C. psittaci* pneumonia. Body temperatures were recorded after administration. The interval from symptom onset to admission and that from symptom onset to diagnosis was recorded.

## 3 Results

### 3.1 General data of patients

The six patients enrolled in this study comprised four males and two females, with a median age of 65.5 years (range, 51–69 years). No patient reported a history of respiratory disease. Four patients had a history of combined hypertension, one had a history of hepatitis A, one had a history of gout, and two patients were immunocompromised (one with carcinoma *in situ* of the colon and one with thymoma). Four patients demonstrated a clear history of exposure to birds (**Table 1**).

**Table 1:** Baseline characteristics and clinical manifestations of patients with Chlamydia psittaci pneumonia.

Case	Sex	Age	Occupation	Past history	Contact history	Clinical manifestation			
						Temperature peak	Initial symptoms	Concomitant symptoms	Lung auscultation
1	Male	68	Retired staff	No	No	39.5	Fever; headache	Chilly sensation; cough; blood-tinged sputum	Coarse breath sounds; no significant rhonchi and moist rales were heard
2	Female	69	Farmer	Open reduction and internal fixation of lumbar vertebrae; syndesmopexy of the left knee	Poultry (chicken)	39	Fever	Chest discomfort and tachypnea; night-sweat; dry cough	Coarse breath sounds; no significant rhonchi and moist rales were heard
3	Male	51	Security guard	Hypertension; hepatitis A; gout; carcinoma in situ of colon	No	39.6	Chest pain; fever	Cough and expectoration; blood-tinged sputum; joint swelling and arthralgia	Coarse breath sounds; moist rales were heard
4	Male	69	Chef	Hypertension; chronic gastritis; thymoma	Poultry (chicken and duck)	40	Fever	Chilly sensation and rigor; dry cough; fatigue; night-swear	Coarse breath sounds; moist rales were heard
5	Male	63	Farmer	Hypertension	Poultry (chicken)	40	Cough and expectoration; fever	Chilly sensation and rigor; fatigue; poor appetite	Coarse breath sounds; no significant rhonchi and moist rales were heard
6	Female	58	Farmer	Hypertension	Poultry (chicken and duck)	39.8	Fever	Chilly sensation and rigor; dizziness; dry cough	Coarse breath sounds; moist rales were heard

### 3.2 Clinical manifestations

All six patients developed fever, with a maximum body temperature of 39.0-40.0 °C reported in four patients and ≥40.0 °C observed in two patients. Five patients reported the development of cough, including dry cough (3/6) and blood-tinged sputum (2/6). Accompanying symptoms included headache (1/6), chilly sensation (4/6), rigor (3/6), night sweats (2/6), chest discomfort and tachypnea (1/6), chest pain (1/6), fatigue (2/6), poor appetite (1/6), and joint swelling and arthralgia (1/6). All patients reported the absence of nasal obstruction, pharyngodynia, muscular soreness, abdominal pain, diarrhea, nausea, or vomiting. Moist rales were heard in three of the six patients, without signs of combined cyanosis, barrel chest, wheezing phlegm, wheezing rale, or pleural friction rubs (**Table 1**).

### 3.3 Laboratory indices

Five out of six patients showed an elevated WBC count and elevated neutrophil percentage. The levels of CRP, PCT, and ESR were increased in all patients and were recorded in the ranges of 78.70-274.26 mg/L, 0.114-3.050 ng/ml, and 36-95 mm/h, respectively, during initial consultations. ALT levels in all six patients were within the normal range; an elevated AST level was present in 2/6 patients. Elevated LDH levels were recorded in three cases in the range of 269-369 U/L at initial consultations. An elevated CK level was present in 1/6 patients; the other patients showed levels within the normal range. After one week of empirical anti-infective therapy, a substantial decrease in CRP and PCT levels was observed in all cases. CRP levels and PCT levels were reduced to those within the normal range in 2/6 patients and 4/6 patients, respectively (**Table 2**).

**Table 2:** Laboratory and imaging findings in patients with Chlamydia psittaci pneumonia.

Laboratory examination											Imaging test		
WBC	NE	hCRP	PCT	ESR	ALT	AST(U/L)	LDH	CK	hCRP (mg/L) at 1 week after treatment	PCT	Diseased region	Lesion pattern	Lesion size (mm*mm)
(*10^9/L)	(%)	(mg/L)	(ng/ml)	(mm/h)	(U/L)		(U/L)	(U/L)		(ng/ml) at 1 week after treatment			
5.29	88.3a	274.26a	3.05a	73a	32	29	164	101	81.22a	0.225a	Right upper lung	Patchy high-density shadow; pleural effusion	55*35
8.76	89.4a	79a	0.221a	36a	32	35	242	63	10.44a	<0.02	Left upper lung, left lower lung	Patchy and mass high-density shadow; air bronchogram sign	39*23
12.17a	83a	91.86a	0.114a	82a	21	25	155	193	8.67a	<0.02	Bilateral multiple lung lesions	Patchy and mass high-density shadow; local consolidation; air bronchogram sign; pleural effusion	67*44
5	81.8a	122.06a	0.227a	53a	28	43a	269a	407a	6.61	0.043	Right lower lung	Patchy high-density shadow; local consolidation; air bronchogram sign	88*56
4.78	61.8	78.7a	0.528a	87a	34	45a	369a	62	4.3	0.056a	Left upper lung	Large patchy high-density shadow; local consolidation; air bronchogram sign; pleural effusion	56*49
7.52	88.5a	241a	0.282a	95a	26	34	286a	178	9.39a	0.041	Left upper lung	Mass high-density shadow with consolidation; air bronchogram sign	57*60

WBC, white blood cell count (normal range 3.50-9.50*10^9/L), NE%, neutrophil percentage (normal range 40.00-75.00%), hCRP, hypersensitive C-reactive protein (normal range 0.00-8.00 mg/L), PCT, procalcitonin (normal range 0-0.050 ng/ml), ESR, erythrocyte sedimentation rate (normal range 0-20 mm/h), ALT, alanine aminotransferase (normal range 7-40 U/L), AST, aspartate aminotransferase (normal range 13-35 U/L), LDH, lactate dehydrogenase (normal range 120-250 U/L), CK, creatine kinase (normal range 40-200 U/L).

### 3.4 Imaging findings

**Figure 1:**
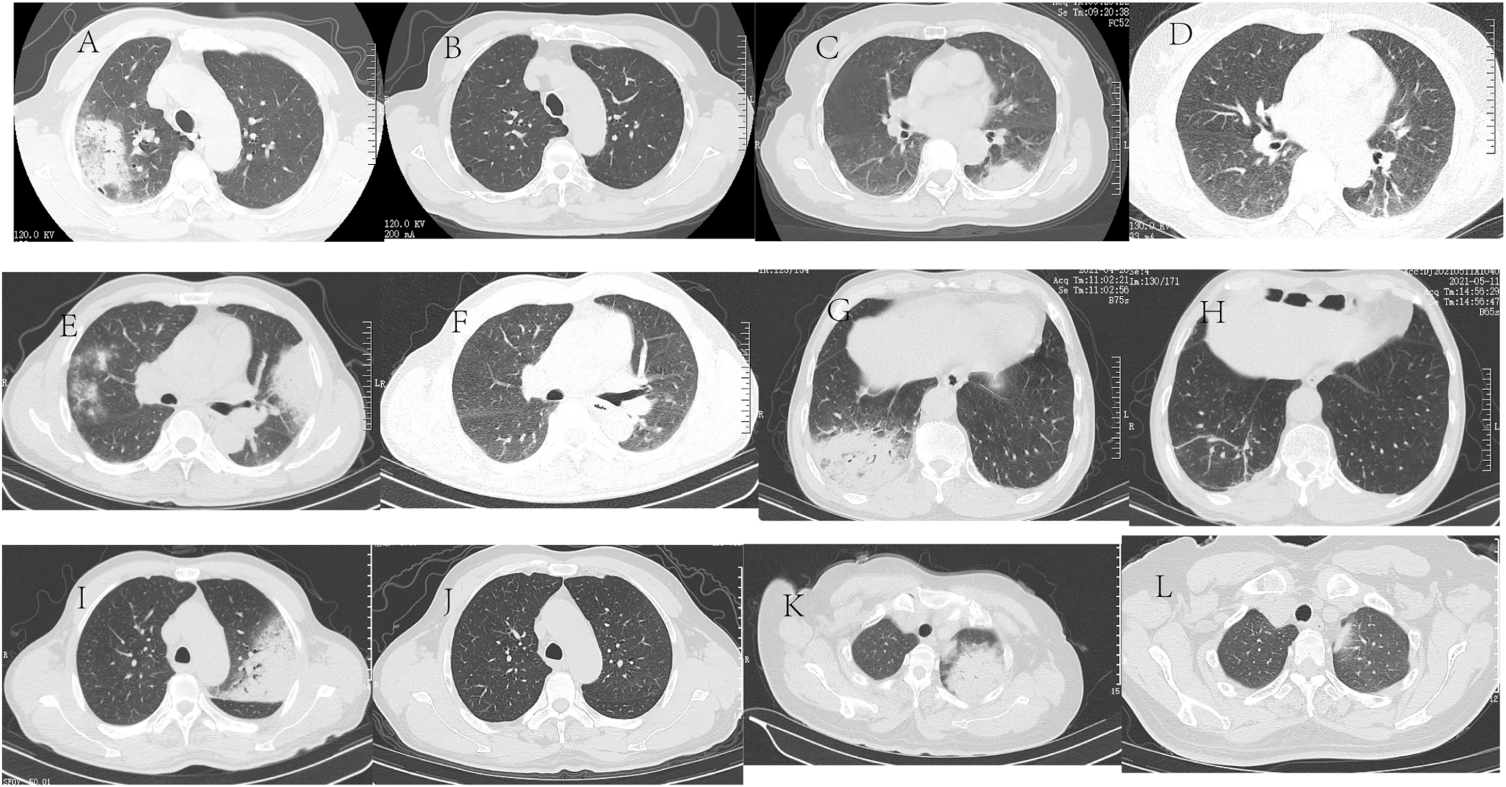
Chest CT of six patients with *C psittaci* pneumonia Chest CT of six patients with *C psittaci* pneumonia. Case 1: A 68-year-old male was admitted because of a fever and headache for three days. **(A)** Chest CT shows a patchy high-density shadow subpleural region in the right upper lung, with a small amount of pleural effusion in the right pleural cavity. **(B)** Two months after discharge from the hospital, chest CT showed that the inflammation in the upper lobe of the right lung almost disappeared. Case 2: A 69-year-old female was admitted because of a fever for two days. **(C)** Chest CT shows patchy and mass high-density shadow in the upper and lower lobes of the left lung, with a sign of an air bronchogram. **(D)** Two months after discharge from the hospital, chest CT showed that the lesions in the upper and lower lobes of the left lung almost disappeared. Case 3, A 51-year-old male was admitted because of left-sided chest pain with a fever for three days. **(E)** Chest CT shows bilateral multiple patchy and mass high-density shadows, accompanied by local consolidation and air bronchogram sign with a small amount of bilateral pleural effusion. **(F)** One month after discharge from the hospital, chest CT showed that the inflammation in the bilateral lungs disappeared. Case 4: A 69-year-old male was admitted because of a fever for seven days. **(G)** Chest CT shows a patchy high-density shadow in the lower lobe of the right lung, with local consolidation and air bronchogram sign. **(H)** One month after discharge from the hospital, chest CT showed that the inflammation in the lower lobe of the right lung disappeared. Case 5: A 63-year-old male was admitted because of a cough and fever for ten days. **(I)** Chest CT shows a large patchy high-density shadow in the left upper lung, with local consolidation; A few consolidation shadows in the left lower lung and a small amount of fluid in the left pleural cavity. **(J)** Two months after discharge from the hospital, chest CT showed that the inflammation in the upper lobe of the left lung almost disappeared. Case 6<nbsp/>A 58-year-old female was admitted because of a fever for four days. **(K)** Chest CT shows a mass high-density shadow in the left upper lung, with consolidation and air bronchogram sign. **(L)** One month after discharge from the hospital, chest CT showed that the inflammation in the upper lobe of the left lung disappeared.

All patients underwent chest CT examinations upon admission. All lung lesions were extensive, with a maximum diameter of 39-88<nbsp/>mm and median diameter of 56.5<nbsp/>mm. Five out of six patients showed the lesions involving unilateral lung, and 1/6 patients had lesions with bilateral lung involvement. The lesions showed patchy high-density exudation shadows with local consolidation, accompanied by air bronchogram signs (5/6) and pleural effusion (3/6) (**Table 2** and **Figure 1**).

### 3.5 Pathogen examination

BALF samples from six patients with suspected *C. psittaci* pneumonia were obtained by bronchoscopy and sent to Hangzhou More BioMedical for mNGS to detect *C. psittaci*. *C. psittaci* with cytomegalovirus was detected in one case (**Table 3**).

**Table 3:** Treatment and outcome of patients with Chlamydia psittaci pneumonia.

Case	Anti-infective drugs used before diagnosis	Time from symptom onset to admission	Time from symptom onset to diagnosis	mNGS results	Anti-infective drugs used after diagnosis
1	Cephalosporin, piperacillin-tazobactam, oseltamivir phosphate, meropenem			Chlamydia psittaci 10, Malassezia 1644, Candida albicans 281	Doxycycline, Meropenem
	
		3d	14d		
2	Piperacillin sodium and sulbactam sodium, levofloxacin			Chlamydia psittaci 152	Doxycycline, Piperacillin sodium and sulbactam sodium
	
		2d	9d		
3	Piperacillin sodium and sulbactam sodium, levofloxacin			Chlamydia psittaci 2	Doxycycline
	
		3d	8d		
4	Cefuroxime, isepamicin sulfate, oseltamivir, piperacillin sodium and sulbactam sodium, levofloxacin, imipenem/cilastatin			Chlamydia psittaci 10, Cytomegalovirus 28	Doxycycline, Imipenem/cilastatin
	
	
	
	
		7d	13d		
5	Cefoperazone-sulbactam, moxifloxacin; imipenem/cilastatin			Chlamydia psittaci 143, Prevotella melaninogenica11	Doxycycline, Imipenem/cilastatin
	
		10d	17d		
6	Ceftazidime, levofloxacin, Piperacillin sodium and sulbactam sodium			Chlamydia psittaci 32, Klebsiella pneumoniae 44	Doxycycline, Piperacillin sodium and sulbactam sodium
	
		4d	14d		

mNGS, metagenomic next-generation sequencing; The results were provided by Hangzhou More Bio Medical.

### 3.6 Treatment and outcomes

All patients received empirical anti-infective therapy prior to a definitive diagnosis, with two-five antibiotics in succession. Two out of six patients were treated with concomitant oseltamivir antiviral therapy because viral pneumonia could not be excluded. After one week of anti-infective therapy, one patient continued to report fever, and five patients reported a reduction of body temperatures back to those within the normal range. However, all six patients did not show substantial improvement in non-specific symptoms, such as cough and expectoration. The time from symptom onset to diagnosis was 8-17 days among six patients, with a median duration of 13.5 days. After diagnosis, as the immediate adjustment of the regimen to doxycycline-based anti-infective therapy, all patients had favorable prognoses and were discharged. All patients underwent chest CT within 1-2 months after treatment, which suggested that the inflammatory lesions had almost disappeared (**Table 3** and **Figure 1**).

## 4 Discussion

*C. psittaci* pneumonia is caused by infection with the pathogen *C. psittaci*, an obligate intracellular gram-negative bacterium that can survive *in vitro (* Guedel et al., 1990). Humans can be infected *via* inhalation of aerosols contaminated with urine, feces, or other excreta from infected birds. Human-to-human transmission is possible but rare. Other potential transmission routes, such as contact with flocks of city pigeons, have been reported but are uncommon (Bourne et al., 2003). In this study, four patients had a clear history of bird exposure, which supports the hypothesis of earlier studies that over 50% of patients with *C. psittaci* pneumonia have a history of bird exposure. The findings highlight the importance of a detailed consultation to identify the disease as early as possible, thereby providing effective treatment.

In this study, two of the cases were immunocompromised and the remaining four were immunocompetent, without substantial specificity in the signs and symptoms of all patients, suggesting no apparent association between the presence or absence of an immunocompromised state and the severity of clinical manifestations. Our data provide further evidence in support of previous reports that *C. psittaci* is not a common infection in immunocompromised populations, and hence, does not pose a significant threat to these individuals (Fang et al., 2021).

The incubation period for *C. psittaci* pneumonia is approximately 5-14 days. Its clinical symptoms are diverse and vary in severity, ranging from mild influenza-like symptoms to systemic symptoms dominated by atypical pneumonia (Fang et al., 2021). In this study, all patients presented with hyperpyrexia (temperature ≥39.0°C), and other symptoms such as cough and expectoration were non-specific. No influenza-like symptoms, such as nasal obstruction and discharge, or myalgia were observed, and lung auscultation lacked specificity. One patient presented with joint swelling, arthralgia, and a history of gout. According to previous reports, *C. psittaci* infections act as a trigger for the development of acute gout in approximately 4% of patients (Su et al., 2021), and in this case, *C. psittaci* infection was considered to cause gout.

In this study, the admission hCRP and ESR levels were substantially elevated in all patients, and the admission PCT level was slightly elevated in general. After one week of empirical anti-infective therapy, hCRP, ESR, and PCT levels were significantly reduced in all patients, but no significant improvement was observed in clinical symptoms and imaging manifestations. Instead, an exacerbation of the disease was observed. Thereafter, two possibilities were considered: i. hCRP, ESR, and PCT had little diagnostic value for *C. psittaci*, and ii. There was a possibility of co-infection with other pathogens.

Three patients were admitted with a short onset, mostly 2-3 days; however, all patients showed extensive pulmonary lesions. Middle-aged or elderly patients with underlying diseases were at risk of severe pneumonia because of rapid disease progression. The progression of *C. psittaci* pneumonia may be associated with old age, underlying diseases (especially cardiopulmonary disease), smoking, nutrition, and timely diagnosis and treatment (Fang et al., 2021). Imaging findings illustrated that pulmonary lesions in six cases mainly involved the unilateral lung. All chest CT scans showed patchy high-density exudation shadows with local consolidation and air bronchogram signs, mostly accompanied by pleural effusion, which is in accordance with previous findings. After treatment, the disappearance of pulmonary lesions was observed in the absence of residual fibrous cord-like exudation, suggesting a good outcome.

With regard to the pathogenic examination, sputum cultures were negative in all patients. PCR is the most specific and rapid method to genotype positive strains and is of paramount importance to identify the source of infection; however, sensitivity is observed only during the acute phase of infection and is rarely applied in clinical practice (Nieuwenhuizen et al., 2018; Lei et al., 2020; Zhang et al., 2021). *C. psittaci* infections are often overlooked, resulting in a lack of timely diagnoses. Owing to the deficiencies of traditional methods, mNGS is an increasingly popular alternative for clinical diagnosis (Nieuwenhuizen et al., 2018; Lei et al., 2020; Zhang et al., 2021). Unknown infection sources can be detected by mNGS, and the relative abundance of pathogens can be quantified. It has the potential to assist doctors in the diagnosis and treatment of mixed infections. In this study, *C. psittaci* was detected in the BALF samples collected from all patients, with unique sequence/reads <<nbsp/>15 in three cases, as tested by the mNGS report. Two reasons were considered, which are outlined as follows: C. psittaci is an intracellular pathogen, and multiple antibiotic treatments had been administered before BALF samples were sent for examination. Cytomegalovirus was also detected by mNGS in one patient with a history of underlying thymoma. It causes latent infection, and the retained virus can be activated in immunocompromised patients (Zhang Z. et al., 2022).

The mNGS results alone are insufficient to underpin the final diagnosis, which can be confirmed by a combination of medical history, clinical symptoms, laboratory examination, and imaging findings. Clinical symptoms, laboratory findings, and imaging findings of the patients lacked specificity. Routine culture and serological tests yielded no positive results. After one course of empirical anti-infective therapy, imaging findings revealed unsatisfactory disappearance of lesions, and BALF was then subjected to mNGS to identify the pathogen. However, the late intervention of mNGS led to a delayed diagnosis, with a time from symptom onset to diagnosis of approximately two weeks. In recent years, retaining respiratory specimens for mNGS testing has been highly recommended for patients with respiratory infections if the pathogen is not clearly defined by routine laboratory tests within three days, and empirical anti-infective therapy is ineffective (Li et al., 2022).

With the identification of noninfectious lesions, a variety of antibiotics were administered prior to diagnosis. However, these were ineffective due to the low sensitivity of *C. psittaci* to these antibiotics and large lesions. All patients were administered antibiotics that were not sufficiently effective. In line with the results of recommended antimicrobial therapies, all patients took a favorable turn following the adjustment to a doxycycline-based anti-infective regimen administered for 10-14 days after diagnosis (Shen et al., 2021). Doxycycline or minocycline is preferred for the treatment of *C. psittaci* pneumonia, followed by azithromycin, clarithromycin, erythromycin, and chloramphenicol, and the antibiotics are administered for a minimum of ten days (Wen et al., 2021).

In conclusion, notwithstanding the complexity, variety, and non-specificity of clinical manifestations in the context of *C. psittaci* pneumonia, certain general clinical characteristics observed in patients are listed as follows: a history of bird exposure; hyperpyrexia with a temperature ≥39.0°C, accompanied by cough; normal WBC count, with elevated neutrophil percentage and mildly elevated hCRP and PCT levels; imaging findings of lesions mostly showing patchy high-density exudation shadows with local consolidation; difficulties in routine pathogen examination and culture, with the confirmation of diagnosis by mNGS; sensitivity to tetracyclines (doxycycline); complete disappearance of the lesion; and good outcome.

## Ethics statement

Written informed consent was obtained for the publication of this case report.

## Author contributions

JD dealt with the case and drafted the manuscript. XuL, JM and XiL assisted collected case data and literature and carried out all the documentary and article work out. WM gave some constructive suggestions for this paper during the revision and production period. HW and JJ contributed to conception, design, and critically revised the manuscript. All authors contributed to the article and approved the submitted version.
